# Replacing soil with waste gangue for the ecological remediation of mining areas facilitated by plant-promoting microorganisms and porous materials

**DOI:** 10.1038/s41598-026-38682-6

**Published:** 2026-02-09

**Authors:** Bo Zhang, Dong Ma, Xingxing Zhou, Lingmei Li, Li Li, Guangsheng Qian

**Affiliations:** 1 College of Chemical and Environmental Engineering, Shiyan Key Laboratory of Biological Resources and Eco-Environmental Protection, Hanjiang Normal University, Shiyan, 442000 People’s Republic of China; 2 Department of Ocean Science and Technology, Faculty of Science and Technology, University of Macau, Macau, 999078 People’s Republic of China; 3 School of Resources and Civil Engineering, Northeastern University, Shenyang, 110819 People’s Republic of China; 4 Datang Environmental Protection Technology Research Institute, Datang Environment Industry Group Co., Ltd., Beijing, 100097 People’s Republic of China; 5 College of Life Science, Shenyang Normal University, Shenyang, 110034 People’s Republic of China

**Keywords:** Mining restoration, Gangue utilization, Tailings substrate, Pot experiment, Ecology, Ecology, Environmental sciences, Microbiology, Plant sciences

## Abstract

Coal-mining byproducts like coal gangue (CG), green mudstone (GC), and oil shale (OS) pose significant risks to the ecological environment and social safety. In this study, waste gangue was treated in situ without supplementary soil addition. Moreover, the effects of plant growth substrates prepared by incorporating chicken manure (+), plant growth-promoting microorganisms (+M), and porous materials (+MM) on soil nutrients, plant growth, and microbial communities were investigated. Compared with untreated gangue, organic matter, total nitrogen, total phosphorus, available nitrogen, and available phosphorus in the CG + MM, OS + MM, and GC + MM groups increased by 170%, 14%, 93%, 174%, 1951%; 51%, 15%, 262%, 147%, 480%; and 230%, 23%, 85%, 78%, 1,186%, respectively. Regarding plant growth, CG had no significant effect on *Lolium* growth, whereas GC and OS drastically decreased *Lolium* biomass. However, the addition of exogenous substances promoted *Lolium* growth. Microbial community network analysis confirmed the presence of *Bdellovibrio*, *Halomonas*, *Nocardioides*, *Sphingobacterium*, *Streptomyces*, and *WD2101 soil groups* in the CG group; *Bdellovibrio*, *Chloroplast*, *Lysobacter*, *Methylophaga*, and *WD2101 soil groups* in the GC group; and *Antarcticibacterium*, *Chloroplast*, *Methylophaga*, *Paracoccus*, *Pontibacter*, and *Rhodonellum* in the OS group. All microbes played key roles in maintaining the microbial community structure. These findings provide novel insights into the ecological restoration of gangue.

**Supplementary Information:**

The online version contains supplementary material available at 10.1038/s41598-026-38682-6.

## Introduction

Coal mining has played a key role in global economic development, contributing to social progress and substantially improving human living standards. Furthermore, it remains strategically important in the global energy consumption system^1^. According to the International Energy Agency, global coal production reached a record high of 9.068 billion tons in 2024, representing a 0.8% year-on-year increase from 8.993 billion tons in 2023. This increase in coal production has led to the excessive accumulation of its industrial byproducts^2^. Coal gangue (CG) is the largest solid waste discharged during coal mining and processing, accounting for approximately 10–20% of total coal production. The current global accumulation of CG is estimated to exceed 10.7 billion tons^3^. Such a large amount of CG not only requires a large surface near the mine leading to land waste but also poses serious threats to the environment and the safety of life and property through weathering and leaching^4^. Similar solid wastes include low-quality oil shale (OS) and green mudstone (GC)^5^. The realization of large-scale comprehensive utilization of waste gangue resources in mining is an urgent task to reduce its negative impact on the ecological environment.

Waste gangue can be mainly used in the following two manners. First, it can be used in building materials, highway construction, landfills, and greening of underground mining by easy industrialization and consumption of a large amount of waste gangue^6–8^; second, it can be used for power generation and heating, as well as for the extraction and synthesis of high value-added products (e.g., zeolites, adsorbents, and flocculants)^9,10^. However, the current utilization rate and quality of CG remain insufficient, and disposal capacity and scale do not meet the requirements of global low-carbon and environmental protection goals^11^. The ecological restoration of mines is an important objective of global environmental protection efforts. The surface layer of weathered CG can be directly planted without soil covering and reclaimed as farmland^12^. Compared with the above mentioned methods of gangue utilization, this approach can not only recycle waste but also improve plant productivity and restore vegetation in mine wastelands^13^. Nonetheless, waste gangue has poor structural stability and water-retention capacity due to its large particle size and extremely low capillary porosity, making it unfavorable for plant growth and development. Previous studies have confirmed that traditional in situ improvement techniques (such as soil covering, addition of conventional organic matter, or chemical fertilizers) often focus on providing short-term physical support or nutrients for plant growth. Their improvement effects are relatively limited and unsustainable while simultaneously increasing industrial costs^14^. Therefore, the addition of suitable exogenous substances is necessary to improve the physicochemical properties of waste gangue^15^.

Currently, exogenous substances used for mine gangue remediation include water retention agents, biochar, and slow-release fertilizers^13^. Porous materials possess unique pore structures and water-retention mechanisms, with potential applications in agriculture, construction, ecological restoration, and other fields^16,17^. For mine restoration, porous materials can improve the soil structure and water-retention capacity of mine gangue. Their pore channels facilitate plant root penetration, provide a favorable habitat for soil microbial communities, and enhance nutrient cycling within the soil environment of mining areas^18^. Previous studies have confirmed that increased soil porosity in tailings is a key indicator of successful ecological restoration in mining areas^19^. Plant growth-promoting microorganisms (PGPMs) are potential microbial resources that can effectively colonize and interact with plant root systems. They promote plant growth and enhance plant self-defense through various mechanisms, such as regulation of soil nutrient availability, inhibition of pathogenic bacteria, and modulation of phytohormone levels^20^. Consequently, such microorganisms have garnered significant attention in the field of sustainable agriculture^20,21^. Moreover, soil microbial communities play a vital role in nutrient cycling, metabolic decomposition, and ecosystem function maintenance. Based on these considerations, combining porous materials with PGPMs as exogenous additives can be effectively applied to waste gangue for ecological restoration of the mine site. However, the improvement effect of porous materials and PGPMs on different waste gangue in mining has not been confirmed yet; thus, the hypothesis must be thoroughly tested to fully understand the effects of adding exogenous substances to waste gangue in mining areas on soil physicochemical indicators, plant growth, and the response mechanism of microbial community changes.

To address the aforementioned issues, waste gangue (CG, OS, and GC) was treated using fertilizers, microbial supplements, and porous materials to prepare planting substrates. Changes in substrate physicochemical indicators, microbial community composition, and plant growth indicators were subsequently analyzed. This study aimed to (1) validate the feasibility of preparing plant growth substrates from waste gangue, (2) clarify the effects of exogenous additives on substrate physicochemical properties and plant growth, and (3) define the response of soil microbial communities to the addition of exogenous substances to the substrate and evaluate the overall metabolic level of the substrates. This study will provide theoretical guidance for the ecological restoration of gangue in mining and provide a basis for the large-scale implementation and application of the new technology.

## Results and discussion

### Physicochemical properties of the substrates

**Fig. 1:**
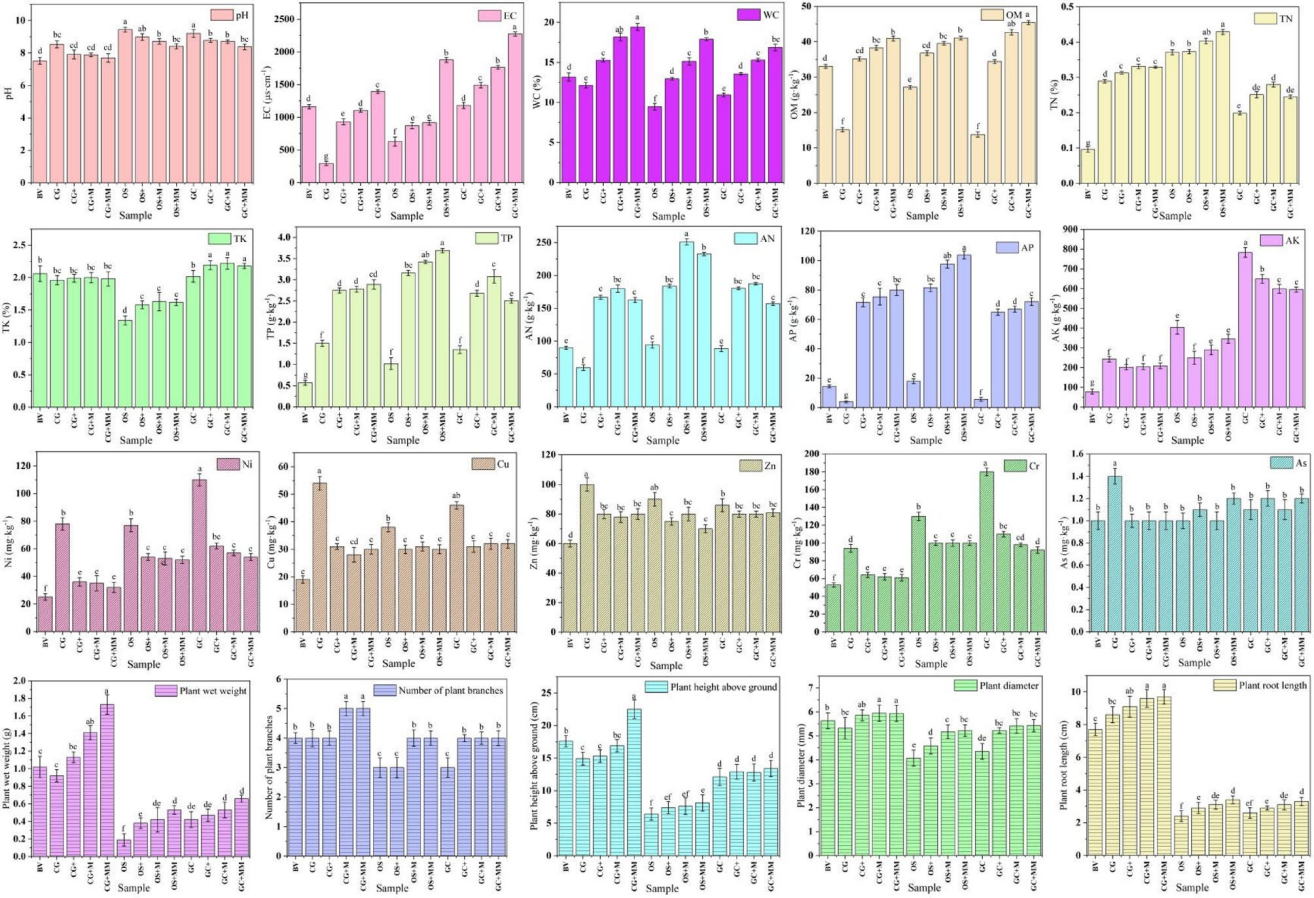
Effects of distinct exogenous amendments on physicochemical indexes of substrate and growth indexes of *Lolium*.

The physicochemical indices of the substrates can be influenced by the nature of the gangue itself and exogenous additives (Fig. 1). In this study, high-pH and high-electrical conductivity (EC) soil (campus soil [BV]), with a pH of 7.51 and an EC of 1162 μs·cm^−1^, was used as the control. Soil pH and EC are important factors in ecosystem fertility formation and evolution^22^. In substrates without exogenous additives, pH values were higher (> 8.5, all strongly alkaline soils), whereas EC values were lower, corresponding to lightly to moderately saline soils in CG and OS and heavy saline soils in GC. After the addition of exogenous substances, substrate pH decreased, whereas EC increased. Previous studies have shown that a pH shift of 1.5 units can reduce soil microbial growth by 50%, which would significantly slow the natural recovery process of mine-affected soils^23^. The pH of the CG + MM treatment was maintained at 7.69 (alkaline soil), which is more favorable for plant root development and nutrient uptake^24^, confirming the improvement of soil ecological conditions. The addition of porous materials substantially increased the substrate EC values, which reached 2274 and 1878 μs cm^−1^ for GC + MM and OS + MM, respectively. Therefore, the optimal dosage of porous materials requires further investigation. The EC values of CG + M and OS + M did not differ significantly from that of BV, whereas the EC of GC (1179 μs cm^−1^) was further enhanced by the addition of exogenous substances, reaching 2,274 μs·cm^−1^ in GC + MM. This increase can be attributed to the high specific surface area and porosity of the porous material, which enhance water absorption and retention, accelerate the mineralization of organic matter (OM), and promote the release of soluble salinity ions, thereby increasing EC^25^. Although higher EC can increase nutrient availability, excessive salinity may negatively impact plant growth^26^; thus, the overuse of porous materials should be restricted. The water content (WC) of GC and OS was much lower than that of BV (13.17%), whereas WC values in GC + and OS + treatments were similar to that of BV. The addition of PGPMs and porous materials further enhanced WC across all substrates, with an overall trend of CG > GC > OS.

The addition of exogenous substances improved OM, total nitrogen (TN), total phosphorus (TP), available nitrogen (AN), and available phosphorus (AP) in the substrates. Compared with CG, CG + MM increased by 170%, 14%, 93%, 174%, and 1951%, respectively; compared with OS, OS + MM increased by 51%, 15%, 262%, 147%, and 480%, respectively; and compared with GC, GC + MM increased by 230%, 23%, 85%, 78%, and 1,186%, respectively^27^. The addition of exogenous substances altered the cycling, retention, and transformation of C, N, and P substrates, thereby increasing their availability. Research indicates that the application of external materials containing nutrients and microorganisms promotes the dissolution of phosphorus and potassium^28^. However, it did not affect total potassium (TK) in the substrate, and the available potassium (AK) content decreased accordingly, which may be related to plant growth and absorption. The fertility indicators of the modified gangue substrates, particularly TN, TP, AN, and AP, were superior to those of BV. Overall, the addition of exogenous substances improved the fertility indicators of OS, but a pH of 8.41 threatens its positive effects on ecological recovery.

According to the risk control standard for soil contamination in agricultural land in China (GB 15618–2018), the Ni, Cu, Zn, Cr, and As concentrations did not exceed the risk screening value in any substrates; however, all exceeded the BV, which may pose a potential threat to human health. The addition of PGPMs and porous materials could reduce the amount of potentially toxic elements in the substrates, but lower pH conditions may accelerate the leaching of potentially toxic elements, resulting in increased availability^29^, and long-term attention should be focused on the migration and transformation of Cr. Spatial and temporal variations in soil physicochemical indicators suggest that the ecological restoration process of the mine gangue significantly affects soil nutrient accumulation and partitioning, thereby driving changes in soil ecosystem processes.

### Plant growth characteristics

The growth index of *Lolium* can reflect the effect of the addition of exogenous substances on waste gangue. Figure 1 illustrates the effects of adding an exogenous substance to the substrate on *Lolium* growth and development. CG had no significant effect on *Lolium* growth (relative to the BV), and all the growth indexes of *Lolium* improved after the addition of fertilizers, PGPMs, and porous materials. Plant diameter, root length, height above ground, number of branches, and wet weight increased by 12%, 13%, 51%, 25%, and 88%, respectively, in CG + MM relative to CG. The above-ground height of *Lolium* in the GC was satisfactory (12 cm); however, with a root length of only 2.6 cm, it may be difficult to withstand the harsh environment of the mine. Relative to GC, plant diameter, root length, height above ground, number of branches, and wet weight were elevated by 25%, 26%, 11%, 33%, and 57%, respectively, in GC + MM. Despite the significant enhancement, all were lower than the plant growth indexes in CG + MM. The plants in the OS group had the worst growth indexes, lower than those of the CG and GC groups. Some studies have demonstrated that high EC increases the osmotic pressure of the soil solution, making it difficult for plant roots to absorb water and inhibiting cell elongation, which in turn shortens root length^30^. GC and OS drastically reduced *Lolium* biomass, and it is difficult to achieve natural ecological restoration in oil shale and GC accumulations without human intervention. *Lolium* growth in GC and OS was unsatisfactory even after the addition of exogenous substances due to the high pH and EC of OS and GC; therefore, future studies should aim to decrease the pH and EC or consider proportionally mixing OS and GC with CG before modification. The improvements in plant growth suggest that ecological restoration of mine gangue is significantly effective and that it is feasible to use PGPMs and porous materials as exogenous substances for the amelioration of waste gangue in the mine.

### Properties of microbial communities

**Fig. 2:**
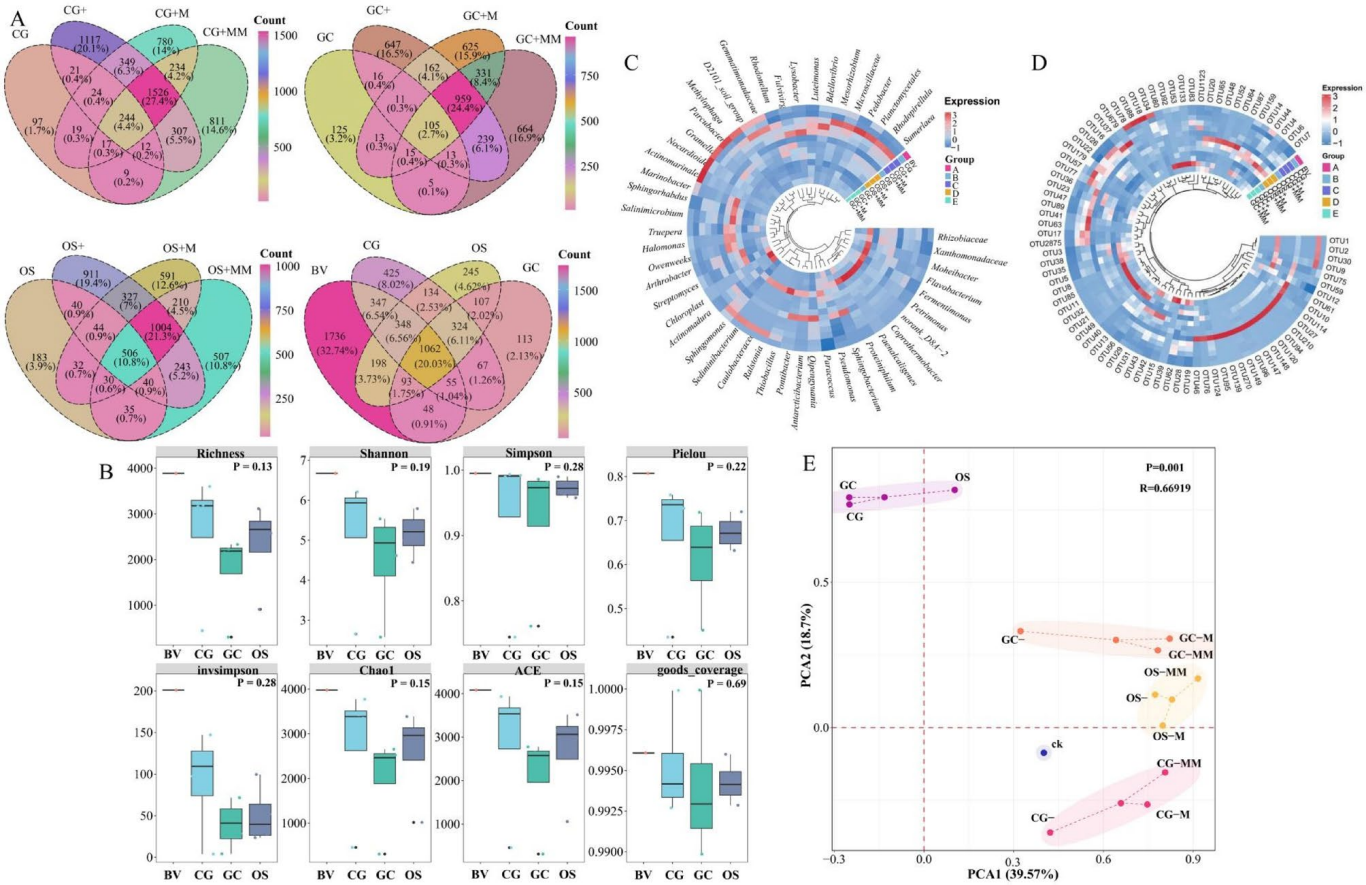
Microbial community structure: Venn diagram (**A**), richness and diversity indicators (**B**), genus-level Circos heatmap (**C**), and OTU-level Circos heatmap (**D**) in the substrate. Principal component analysis of treatments based on Bray–Curtis distances of microbial communities (**E**). *Note* Figures (**C**) and (**D**) show the top 50 genera and top 85 OTUs in relative abundance.

The Venn diagram shows the classification of OTUs (Fig. 2A). The highest OTU classification unit in BV was 3887, the CG group from high to low was CG + (3600) > CG + M > CG + MM > CG (443), the GC group from high to low was GC + MM (2331) > GC + M > GC +  > GC (303), and the OS group from high to low was OS + (3115) > OS + M > OS + MM > OS (910). Microorganisms are not passive responders but active drivers of ecosystem succession, playing a pivotal role in soil systems of mining areas^31^. In this study, microbial communities exhibited relatively low abundances due to the oligotrophic nature of pristine gangue substrates (CG, GC, and OS). The composition and diversity of soil microbial communities are largely influenced by differences in nutrient availability and nutrient preferences^32^. After prolonged natural succession, only some soil microorganisms can adapt to the harsh mining environment, become initially colonized, and influence the ecosystem during their life cycle^33^. The addition of fertilizers substantially increased OTU taxonomic units, whereas the addition of PGPMs and porous materials reduced OTU numbers in CG and OS substrates, possibly related to co-nutrient microorganisms’ ecological niche grabbing^17^. Overall, 1062 co-expression taxa (20% of the total) were found in BV, CG, GC, and OS; unique expression taxa numbers were BV (1736) > CG group (425) > OS group (245) > GC group (113). Coverage indices for all substrates were > 0.99, indicating sufficient sequencing depth to accurately characterize microbial diversity (Fig. 2B). The microbial community abundance indices Chao and ACE were consistent with the observed trend of the OTU taxonomic units. The microbial community diversity index followed a similar pattern; although exogenous substance addition increased diversity, levels remained lower than BV. The diversity index of the microbial community was in the order from high to low, as CG group > OS group > GC group.

Taxonomic analysis showed that approximately 99% of OTUs in the substrates were categorized into the corresponding microbial phylum, obtaining 44 phyla, 136 classes, 345 orders, 610 families, 1300 genus, and 1809 species. The dominant genera in CG, OS, and GC were *Ralstonia*, *norank Caulobacteraceae*, and *Sphingomonas* (Fig. 2C). Previous studies have shown that the plant pathogen *Ralstonia* can proliferate in soil environments and induce plant wilt^34^. It is a highly pathogenic, soil-borne plant pathogen with a broad host range encompassing numerous crops, representing one of the most destructive and difficult-to-eradicate soil-borne diseases^35^. A major obstacle to the ecological restoration of waste gangue in mining is the presence of such phytopathogenic bacteria, which has rarely been reported in the field of mine ecological restoration. *Norank Caulobacteraceae* is tolerant to heavy metals and were dominant genus in the original soil^36^. *Sphingomonas* plays a role in the decomposition and transformation of soil organic carbon, promotes carbon cycling and nutrient release, and can adapt to harsh environments, such as extreme low temperatures, high salinity, or droughts^37^. After the addition of exogenous substances, the relative abundance of *Ralstonia* and *norank Caulobacteraceae* significantly decreased and were no longer among the dominant genera, with a relative abundance decreasing from 70.77 to 0.08% in CG group, from 68.38 to 0.04% in GC group, and from 13.08 to 13.03% in OS group.

Analysis of the OTU level of the substrate microbial community revealed that OTU1 (*Ralstonia pickettii*) and OTU2 (*Caulobacteraceae sp.*) were the dominant genera in CG, GC, and OS substrates (Fig. 2D). In addition, the dominant species of CG was OTU9 (*Sphingomonas leidyi*), which belongs to *Proteobacteria*. *S. leidyi* promotes plant growth by fixing nitrogen, removing phosphorus, and inducing indole acetic acid production^38^. After the addition of exogenous substances, the dominant species of the CG group changed to OTU6 (*Salinimicrobium*), OTU16 (*Rhodopirellula*), OTU17 (*unclassified Luteimonas*), OTU7 (*Sphingorhabdus*), OTU5 (*Fermentimonas caenicola*), and OTU3 (*Xanthomonadaceae*). *Salinimicrobium* is a gram-positive, salt-tolerant, and specialized alkaliphilic bacteria that can utilize a variety of organic compounds, such as sugars, organic acids, and amino acids, as energy sources. It promotes the degradation of hydrocarbons in the soils^27^ and plays an important role in carbon and nutrient cycling in the ecosystem^39^. *Rhodopirellula* can play a role in phosphate solubilization and organic P mineralization^40^, as well as having an inhibitory effect on a soil-borne disease (tobacco black shank disease)^41^. *Unclassified Luteimonas* is a PGPM, salt-tolerant bacteria that promotes peroxidase secretion and C/N/S conversion and can significantly enhance plant inter-root growth and colonization^42^. *Sphingorhabdus* reduces the effectiveness of PTEs and can degrade aromatic and aliphatic hydrocarbons^43^. *Fermentimonas caenicola* has no reported soil function and is a core functional strain associated with efficient biogas production^44^. *Xanthomonadaceae* are common electroactive bacteria that accelerate exogenous electron transfer to facilitate the complexation of iron oxides and dissolved OM^45^, with potentially antagonistic and beneficially interacting microbiota^46^.

The dominant species in OS were OTU10 (*Pseudomonas stutzeri*) and OTU27 (*Antarcticibacterium*). *P. stutzeri* is a denitrifying bacterium that exhibits remarkable adaptability to extreme environments^47^, which explains its high relative abundance (13.16%). It efficiently removes nitrogen under the triple stress of high alkalinity, high salinity, and antibiotic exposure^48^. *Antarcticibacterium* belongs to a genus found in marine sediments with high salinity tolerance^49^; however, its environmental functionality has not yet been reported. The abundance of the dominant species in the OS group significantly decreased after the addition of exogenous substances, and that of OTU4 (*unclassified Marinobacter*), OTU6, OTU3, OTU11 (*Flavobacterium cloacae*), and OTU7 increased, becoming the dominant species in the OS group. Only OTU1 and OTU2 were the dominant species in GC, with a combined relative abundance of 68.38%. After the addition of exogenous substances, the relative abundance of OTU1 and OTU2 combined was < 0.5% and was replaced by OTU5, OTU3, OTU6, OTU10, and OTU7.

The PCA results in Fig. 2E showed that CG, GC, and OS clustered better in the absence of exogenous substances, whereas microbial community structures differed significantly after the addition of exogenous substances. Alterations in the physicochemical conditions of the substrate initiated the reorganization of the microbial community structure and functional succession. These changes contribute to the long-term stability and sustainable recovery of the gangue ecosystem in mining areas.

### Relationship between microbial communities and physicochemical properties of the substrates

**Fig. 3:**
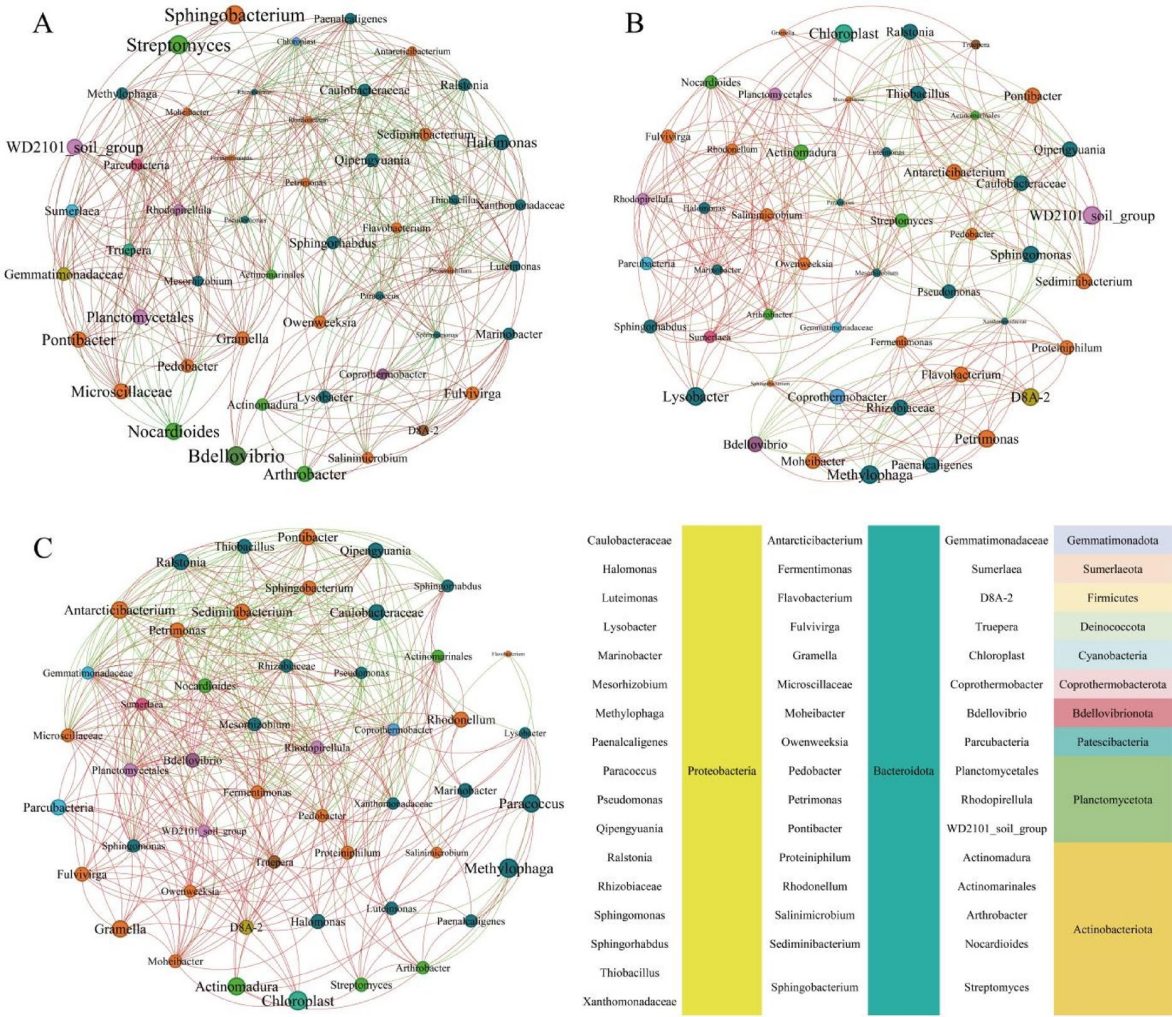
Network analysis of CG (**A**), GC (**B**), and OS (**C**) based on microbial community (at the genus level) and soil physicochemical indicators. *Note* Using data obtained from high-throughput sequencing (selecting the top 50 genera), networks were constructed based on Pearson correlation calculations (gate level: |R|≥ 0.80, genus level: *p* ≤ 0.20) as valid connections.

**Fig. 4:**
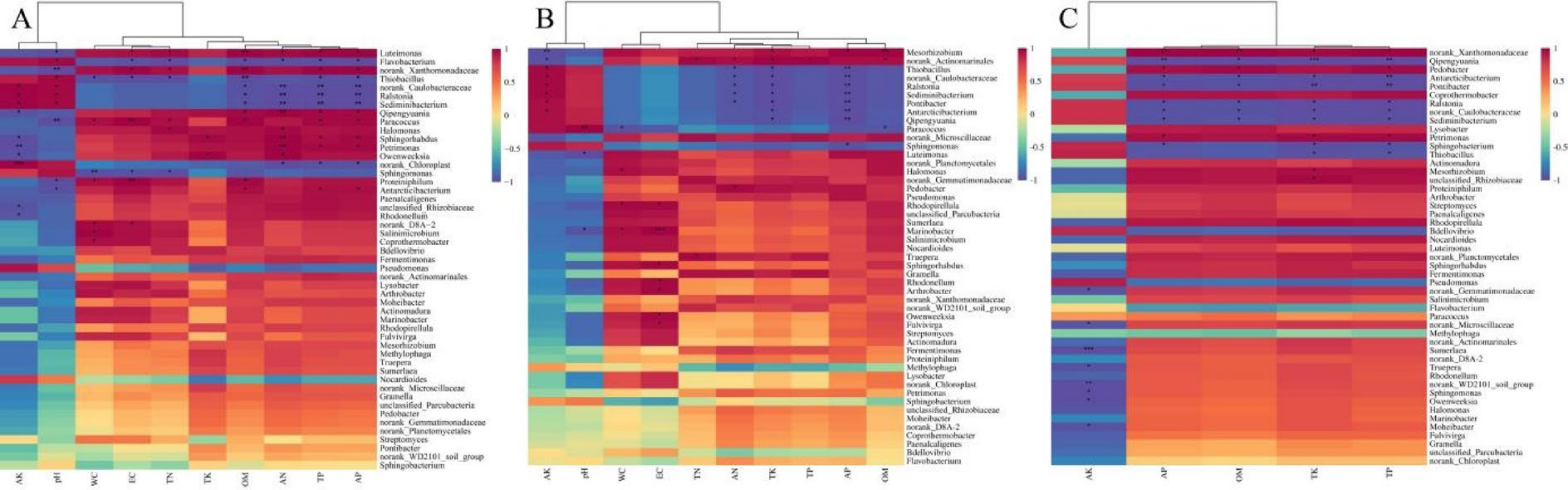
Spearman correlation heatmaps showing the relationships between dominant microbial communities at the genus level and environmental factors. CG, GC, and OS microbial communities-environmental factors (**A**, **B**, **C**). *Note* The Spearman correlation heatmap displays soil physicochemical indicators horizontally and the top 50 microbial community genera-level classifications vertically.* is significant difference at *p* of ≤ 0.05; ** is significant difference at *p* of ≤ 0.01.

Network analysis was used to assess coexisting or possible interactions between intricate populations of soil microorganisms^50^. Figure 3 illustrates network analysis diagrams of microbial communities after addition of exogenous substances to the substrate and Fig. 4 illustrates their correlation with environmental factors and plant growth indicators. Microbial communities in the various gangue substrates responded to physicochemical changes through different mechanisms.

The CG network exhibited the highest connectivity, including 49 nodes and 471 edges, consistent with its high microbial community richness (Fig. 2B); further, pH and EC conditions in CG were more favorable for microbial community survival than those in OS and GC (Fig. 1). Soil pH may restrict the growth of specific microbial groups, while the effectiveness and availability of nutrients in the soil are also closely related to soil pH, which in turn affects the functional characteristics of soil microorganisms^51^. In the CG group, *Bdellovibrio*, *Halomonas*, *Nocardioides*, *Sphingobacterium*, *Streptomyces*, and *WD2101 soil groups* played a key role in maintaining microbial community structure (Fig. 3A). Furthermore, 329 pairs of substrate microbial communities showed positive correlations with physicochemical indices, and 161 pairs showed negative correlations, among which AK, EC, pH, and TP were the main drivers of microbial community (Fig. 4A). *Bdellovibrio* exhibits high potential as biocontrol agent and can prey on a wide range of plant pathogens^52^. This may explain the decrease in the abundance of *Ralstonia* in CG. As this strain is more sensitive to pH, its abundance was higher in CG + M and CG + MM treatments. *Halomonas* is a moderately halophilic bacterium that has beneficial effects as a soil inoculant in moderately saline, arid soils^53^. In this study, it showed a significant positive correlation with AN and TN. *Nocardioides* are involved in the soil nitrogen cycle and are considered as indicator of ecological restoration of saline soils^54^. In this study, their abundance was positively correlated with the addition of exogenous substances. *Sphingobacterium* produces beneficial phytohormones (e.g., gibberellins and indoleacetic acid) that can improve plant growth under potentially toxic elemental stress in drought and saline soils^55^. In this study, *Sphingobacterium* was positively correlated with all associated microorganisms. *Streptomyces* is a low-abundance filamentous bacterium present in soil that plays a crucial role in nutrient cycling and interacts with other microorganisms to enhance soil ecosystem stability and biodiversity^56^. The *WD2101 soil group* is an oligotrophic bacterium specifically associated with certain grass species^57^. In this study, it was positively associated with most dominant genera, but its abundance decreased after the addition of a porous material.

In contrast, GC network had the lowest connectivity, consistent with its microbial community abundance, including only 336 edges with a positive correlation of 71.1%, mainly attributed to the high pH and EC of the GC. In CG group, *Bdellovibrio*, *Chloroplast*, *Lysobacter*, *Methylophaga*, and *WD2101 soil groups* played a key role in maintaining the microbial community structure (Fig. 3B). In GC, 312 pairs of microbial communities were positively correlated with substrate physicochemical indexes and 178 pairs negatively correlated, among which OM, WC, AK, pH, and EC were the main microbial community driver. *Chloroplasts* are soil green algae whose functions are largely unexplored. It can resist short- and long-term salt stress, exhibiting sustained growth, elevated photosynthetic activity, and increased polysaccharide, soluble protein, and total lipid content, thus being considered a key flora for saline soil remediation^48^, where it positively correlated with all relevant microorganisms and EC in this study. *Lysobacter* is a powerful biocontrol bacterium that produces antimicrobial weapons, such as lytic enzymes, toxins, and secondary metabolites to promote plant growth and immune control of plant diseases by directly inhibiting pathogen growth and infection. In this study, the abundance of *Lysobacter* significantly increased with the addition of PGPMs and porous materials, contributing to ecological restoration (Lysobacter enzymogenes: A fully armed biocontrol warrior). *Methylophaga* participates in the degradation of aromatic compounds in soil and is a key participant in methane oxidation, being negatively correlated with all relevant microbial genera in this study^58^.

The network connectivity of the OS group was similar to that of CG, including 462 edges with a positive correlation of 63.8%. *Antarcticibacterium*, *Chloroplast*, *Methylophaga*, *Paracoccus*, *Pontibacter*, and *Rhodonellum* played key roles in maintaining the structure of the microbial community in this group. Among these, 296 pairs of microbial communities were positively correlated with physicochemical indices, and OM, TK, AK, TP, and AP were the main regulators of the microbial community. These microorganisms are defined as the core microbiome of OS. Studying their coexistence characteristics within ecological niches has important implications for ecological restoration in mining areas^59^. *Antarcticibacterium* is known for its salt tolerance and ability to survive under harsh environmental conditions^60^. Its abundance was highest in OS (5.88%) and GC (1.64%), which confirms previous observations. In this study, its abundance was positively correlated with pH and negatively correlated with other fertility indicators. *Paracoccus*, commonly isolated from PAH-contaminated soils, can use fluorescent anthracene, pyrene, and benzo[a]pyrene as sole carbon and energy sources for growth and has potential for bioremediation of PAH-contaminated soils^61^. OS contains a large number of organic components, which explains its high abundance^62^. *Pontibacter* is a beneficial bacterium that can significantly improve soil resistance in saline soils under saline, alkaline, and drought conditions^63^. In this study, it was negatively correlated with fertility indicators such as WC, OM, and AN. Although the function of *Rhodonellum* in soil environment has not been reported, its abundance significantly increased with the addition of exogenous substances and positively correlated with *Actinomarinales*, *Marinobacter*, *Sphingorhabdus*, *Rhizobiaceae*, and the *WD2101 soil group* strains.

### Functional genes related to soil microbial carbon, nitrogen, and phosphorous cycles

**Fig. 5:**
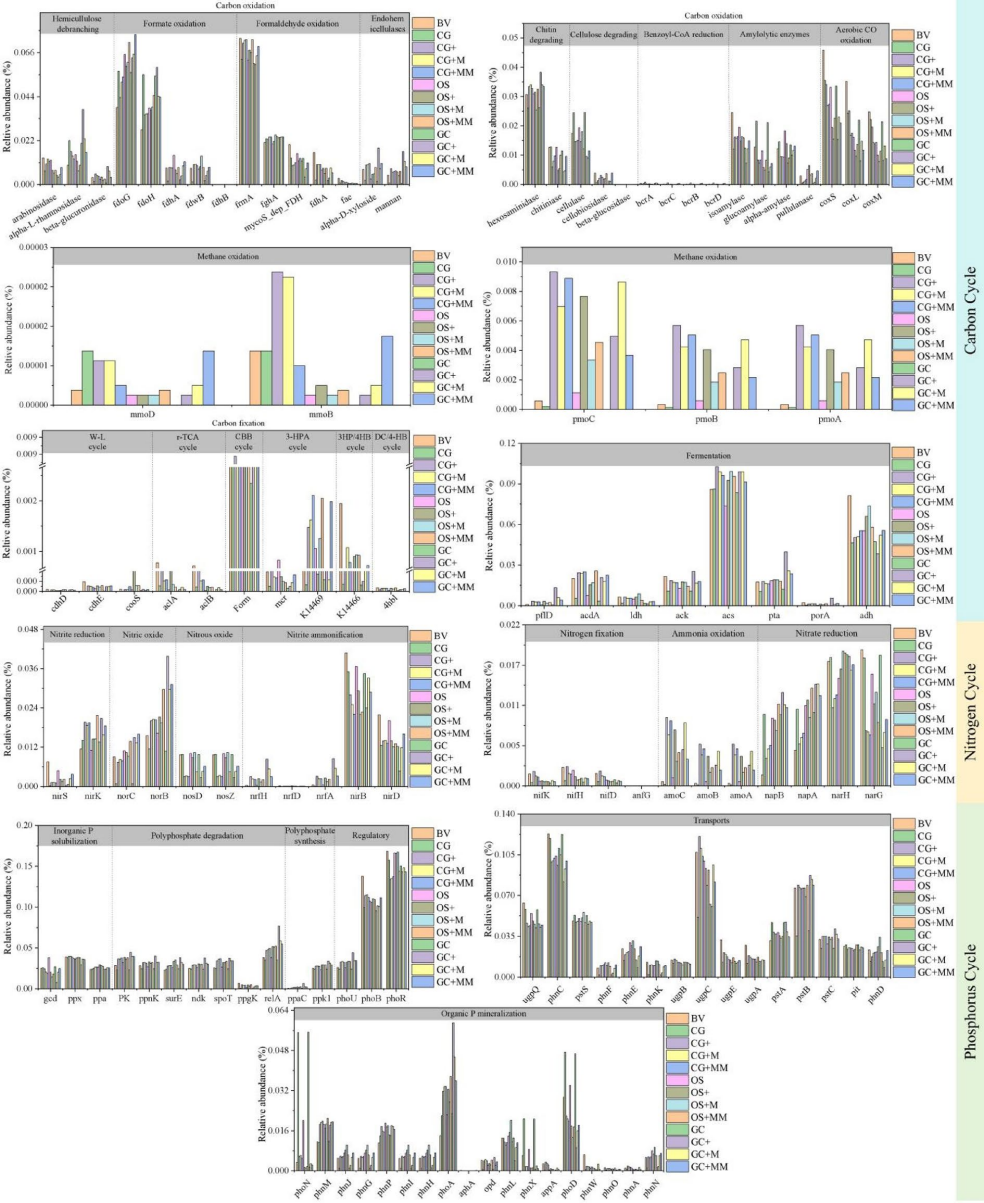
Relative abundance of key functional genes in the carbon, nitrogen, and phosphorus cycles in substrate microbial communities.

The potential functions of microorganisms can be analyzed using 16S rRNA gene data and PICRUSt^64^. A total of 75, 23, and 49 functional gene categories associated with the carbon, nitrogen, and phosphorus cycles were identified using KEGG database annotations (Fig. 5). Organic carbon oxidation plays a major role in the carbon cycle^65^. After the addition of exogenous substances, the abundance of organic carbon oxidation genes in CG, GC, and OS gradually decreased. In contrast, the abundance of carbon fixation, fermentation, and methanotrophy positively correlated with the addition of exogenous substances, being similar to BV; CBB cycle (*Form II*) is dominant in carbon fixation, acetate-to-acetyl-CoA (*acs*), and alcohol utilization (*adh*) in fermentation; and methane oxidation (*pmoA*, *pmoB*, and *pmoC*) is dominant in methanotrophy. This study demonstrated that soil microorganisms in mine waste gangue prefer aerobic pathways (CBB, 3HP, and 3HP4HB) over anaerobic ones (DC4HB, WL, and rTCA) for fixing inorganic carbon^66^. The higher abundance of the CBB cycle suggests adaptability to extreme mining environments and nutrient limitation. Efficient sequestration of carbon accelerates plant biomass accumulation and enhances soil OM^65^.

*nifH* plays a key role in nitrogen fixation in the nitrogen cycle. *nifH* abundance increased (exceeding BV) after adding exogenous substances to CG but decreased in OS and GC. Biological nitrogen fixation is important for ecosystem nitrogen acquisition, and the elevated *nifH* abundance indicated ecological restoration of the CG, while the significantly lower relative abundance in GC and OS could be caused by harsh physicochemical indicators (e.g., high pH and EC) and nutrient deficiency^67^. After addition of exogenous substances, the abundance of *amoC*, *amoB*, and *amoA* in ammonia oxidation increased over that of BV levels, as did the abundance of *norC* and *norB* in nitric oxide reduction, while *nosD* and *nosZ* involved in nitrous oxide reduction decreased. *nirB* and *nirD* were dominant in nitrite ammonification, but their abundance was negatively correlated with the addition of exogenous substances^68^.

In the phosphorus cycle, inorganic phosphorus solubilization is regulated by *gcd*, *ppx*, and *ppa*; their abundance was highest in OS, which was close to that of BV after adding exogenous substances to CG^69^. This result is consistent with the high TP and AP contents observed in OS. After adding PGPMs and porous materials, the abundance of phosphorus-solubilizing microorganisms increased, decreasing phosphorus stress, dissolving mineral-bound phosphorus through the secretion of organic acids, phosphatases, and other mineral-bound phosphorus while interacting with carbon-fixing microorganisms (CBB cycle) to form a synergistic network that accelerates the “carbon–nitrogen-phosphorus” cycling process^70^. As pH is a major factor influencing phosphorous cycling, soil pH improvement had a significant positive effect on TP availability^71^. The abundance of organic phosphorus mineralization in the substrate decreased after adding exogenous substances; *PK* in polyphosphate degradation, *ppnK*, *surE*, *ndk*, *spoT*, *relA*, and *ppk1* in polyphosphate synthesis all play key roles, and their abundance gradually increased after the addition of exogenous substances, much higher than that of BV levels. The increase in abundance was accompanied by proliferation of phosphorus-solubilizing bacteria that secrete citric and oxalic acids to dissolve mineral phosphorus, forming a positive feedback loop of “phosphorus degradation–activation”^72^. The relative abundance of transport increased after the addition of exogenous substances (including phosphonate transport systems and glycerol-3-phosphate transporters), but there was still a gap with BV.

## Conclusions

In this study, we investigated the effects of exogenous substance addition on planting substrates containing waste gangue (CG, GC, and OS) in terms of soil physicochemical properties, PTEs, plant growth performance, and microbial community structure. After adding exogenous substances, the EC, OM, TN, TP, AN, and AP of the gangue substrate increased to various degrees and the pH decreased. According to the risk control standards for soil pollution on agricultural land, Ni, Cu, Zn, Cr, and As contents did not exceed the risk screening values in any substrates. CG had no significant effects on *Lolium* growth (relative to BV), whereas GC and OS drastically reduced *Lolium* biomass. The low microbial community abundance in CG, GC, and OS (CG > OS > GC) improved by adding exogenous substances but was still lower than BV. AK, EC, pH, and TP in the CG group, OM, WC, AK, pH, and EC in the GC group, and OM, TK, AK, TP, and AP in the OS group were the main drivers of microbial communities. The CBB cycle (*Form II*) was dominant in C fixation, acetate-to-acetyl-CoA (*acs*) and alcohol utilization (*adh*) in fermentation, and methane oxidation (*pmoA*, *pmoB*, and *pmoC*) was dominant in methanotrophy. In the N cycle, *nifH* was involved in nitrogen fixation. In the phosphorus cycle, inorganic phosphorus solubilization was regulated by *gcd*, *ppx*, and *ppa*, and their abundance was close to that of BV after adding exogenous substances to CG.

## Materials and Methods

### Study area and collection of gangue samples

Fushun West Open-Pit Mine, Liaoning Province, China, once the largest open-pit coal-mining mine in Asia, was selected as study area (N 41° 50′ 7.29″–N 41° 51′ 11.74″, E 123° 51′ 12.33″–E 123° 55′ 40.85″). In 2018, the mine was closed, and since then, it is undergoing ecological restoration. During the mining period, different types of waste gangue were accumulated, including GC (N 41° 50′ 51.48″, E 123° 55′ 13.11″) and OS (N 41° 50′ 54.46″, E 123° 55′ 10.60″) in the east, and CG in the south (N 41° 50′ 7.52″, E 123° 53′ 13.77″) of the mining area. The “S” method was used to collect shallow (5–20 cm) gangue in GC, OS, and CG accumulation areas. After removing stones, gravel, and leaves, the gangue was placed into a sterile bag, sealed, and transported to the laboratory in a 4 °C storage box.

### Pot experiment design

The pot experiment was conducted in the Environmental Engineering Laboratory of Northeastern University, China. Campus soil randomly collected from Northeastern University served as the control sample (BV). The gangue and campus soil samples were air-dried; pretreated to remove stones, gravel, and leaves; and passed through a 2.00 mm sieve for later use. Table 1 illustrates the substrate treatments and designations. Each substrate weighed approximately 1.00 kg and was loaded into a pot with an outer diameter of 17 cm and a height of 11 cm. Each substrate pot was maintained in triplicate for 3 months, and agitated periodically to retain moisture at field capacity. *Lolium*, a plant with low soil requirements and fast sprouting characteristics, was purchased from Hetian Seed Industry. The fertilizer used was domestic chicken manure. The microbial supplement was a laboratory self-isolation of *Bacillus paramycoides* and *Paenarthrobacter sp*, which exhibit phosphorus solubilization, potassium release, and tolerance to potentially toxic elements^18^. Porous materials were obtained from the Institute of Mineral Genesis of Northeastern University, and previous studies were used to obtain material properties^73^.

**Table 1:** Substrate treatments and designation.

Groups	Substrate name	100 g fertilizer	5% PGPMs	5% porous material
BV	BV	–	–	
CG group	CG	–	–	–
	CG +	√	–	–
	CG + M	√	√	–
	CG + MM	√	√	√
GC group	GC	–	–	–
	GC +	√	–	–
	GC + M	√	√	–
	GC + MM	√	√	√
OS group	OS	–	–	–
	OS +	√	–	–
	OS + M	√	√	–
	OS + MM	√	√	√

The “%” in the table refers to the mass fraction. BV, campus soil substrate; CG, coal gangue substrate, GC, green claystone substrate; OS, oil shale substrate; + , chicken manure added to the substrate; + M, chicken manure and plant growth-promoting microorganisms added to the substrate; and + MM, chicken manure, plant growth-promoting microorganisms and porous material added to the substrate.

### Determination of soil physicochemical properties and plant growth indicators

The WC, pH, and EC of the substrate were determined using a vacuum drying oven (DZF-6050, Shanghai Boxun Industrial Co., Ltd., China), a pH meter (PB-10, Shanghai Weiye Instrument Factory, China), and an EC meter (DDS-307, Shanghai Weiye Instrument Factory, China), respectively. Traditional soil nutrient indicators, including OM, TN, TP, TK, AN, AP, and AK, were determined using previously described methods^74^. Moreover, potentially toxic elements (Ni, Cu, Zn, Cr, and As) were determined through inductively coupled plasma emission spectrometry (Optima 8300 DV, PE Company, USA); all tests were performed according to Chinese national standards (HJ 803-2016)^75,76^. Wet weight, stem diameter, plant height, root length, and the number of *Lolium* branches were measured using a balance (PL203, Mettler-Toledo Instruments GmbH, Switzerland) and a Vernier caliper (DL92200P, Deli Group GmbH, China). Each test set comprised three biological replicates for analytical accuracy.

### High-throughput sequencing

High-throughput sequencing of bacterial 16S rRNA genes was performed on the Illumina MiSeq platform. V3–V4 hypervariable regions of the bacterial 16S rRNA gene were amplified with 341F (CCTACGGGGNGGCWGCAG) and 805R (GACTACHVGGGTATCTAATCC) primers, and the sequencing reads were PE300/PE250. The sequencing work was entrusted to Sangon Biotech (Shanghai) Co., Ltd., and the soil samples were stored in a refrigerator at − 80 °C before sequencing. Sequencing data were deposited in the National Center for Biotechnology Information database under accession number PRJNA1265368. The reaction system for PCR amplification and the subsequent sequencing operation are described in the Supplementary Information.

### Statistical analysis

Raw data (including substrate physicochemical indicators, *Lolium* growth indicators, and microbial community data) were statistically analyzed using IBM SPSS Statistics 22 and Excel 2021. Substrate physicochemical indicators; *Lolium* growth indicators; relative abundance graphs of key functional genes for carbon, nitrogen, and phosphorus cycles were plotted using Origin Pro 9.1; and composite graphs were constructed using PowerPoint 2021. The OmicStudio online bioinformatics platform toolkit (https://www.omicstudio.cn/home) was used to draw Venn diagrams, Circos heatmaps, principal component analysis graphics, Spearman correlation heatmaps, and redundancy analysis graphics. Co-occurrence network visualization was performed using Gephi with the Frucherman–Reingold layout algorithm.

## Supplementary Information

Below is the link to the electronic supplementary material.

Supplementary Material 1

## Data Availability

The data that support the findings of this study are available from the corresponding author (Guangsheng Qian) upon reasonable request.
