# Supplementary material for: Replacing soil with waste gangue for the ecological remediation of mining areas facilitated by plant-promoting microorganisms and porous materials
Source: Sci Rep. 2026 Feb 9;16:7806. doi: 10.1038/s41598-026-38682-6 (PMC12949124; doi:10.1038/s41598-026-38682-6)
Supplement: Supplementary file 1 — Supplementary Material 1 [file 41598_2026_38682_MOESM1_ESM.docx]

Supplementary Material for

Replacing soil with waste gangue for the ecological remediation of mining areas facilitated by plant-promoting microorganisms and porous materials

Bo Zhang ^a, b, c, #^, Dong Ma ^c, #^, Xingxing Zhou ^a^, Lingmei Li ^e^, Li Li ^a^, Guangsheng Qian ^b, d, (🖂)^

^a^ College of Chemical and Environmental Engineering, Shiyan Key Laboratory of Biological Resources and Eco-environmental Protection, Hanjiang Normal University, Shiyan 442000, P.R. China.

^b^ Department of Ocean Science and Technology, Faculty of Science and Technology, University of Macau, Macau, 999078, P. R. China.

^c^ School of Resources and Civil Engineering, Northeastern University, Shenyang, 110819, China.

^d^ Datang Environmental Protection Technology Research Institute, Datang Environment Industry Group Co., Ltd., Beijing, 100097, China.

^e^ College of Life Science, Shenyang Normal University, Shenyang 110034, P. R. China.

# Bo Zhang and Dong Ma contributed equally to this study.

🖂 Corresponding author: guangshengqian@foxmail.com.

**The reaction system for PCR amplification and the subsequent sequencing operation**

Total community genomic DNA extraction was performed using a E.Z.N.A™ Mag Bind Soil DNA Kit (Omega,M5635-02,USA), following the manufacturer's instructions. We measured the concentration of the DNA using a Qubit 4.0 (Thermo, USA) to ensure that adequate amounts of high-quality genomic DNA had been extracted.

Our target was the V3–V4 hypervariable region of the bacterial 16S rRNA gene. PCR was started immediately after the DNA was extracted.The 16S rRNA V3–V4 amplicon was amplified using 2×Hieff® Robust PCR Master Mix (Yeasen, 10105ES03,China). Two universal bacterial 16S rRNA gene amplicon PCR primers (PAGE purified) were used: the amplicon PCR forward primer(CCTACGGGNGGCWGCAG) and amplicon PCR reverse primer (GACTACHVGGGTATCTAATCC). The reaction was set up as follows: microbial DNA (10 ng/µl) 2µl; amplicon PCR forward primer (10 µM) 1µl; amplicon PCR reverse primer (10 µM) 1µl; 2×Hieff® Robust PCR Master Mix (Yeasen, 10105ES03,China) (total 30µl).

The plate was sealed and PCR performed in a thermal instrument (Applied Biosystems 9700, USA) using the following program: 1 cycle of denaturing at 95°C for 3 min, first 5 cycles of denaturing at 95°C for 30 s, annealing at 45°C for 30 s, elongation at 72°C for 30 s, then 20 cycles of denaturing at 95°C for 30 s, annealing at 55°C for 30 s, elongation at 72°C for 30 s and a final extension at 72°C for 5 min. The PCR products were checked using electrophoresis in 2 % (w/v) agarose gels in TBE buffer (Tris, boric acid, EDTA) stained with ethidium bromide (EB) and visualized under UV light.

We used Hieff NGS™ DNA Selection Beads (Yeasen, 10105ES03,China) to purify the free primers and primer dimer species in the amplicon product.Samples were delivered to Sangon BioTech (shanghai) for library construction using universal Illumina adaptor and index. Before sequencing, the DNA concentration of each PCR product was determined using a Qubit® 4.0 Green double-stranded DNA assay and it was quality controlled using a bioanalyzer (Agilent 2100, USA). Depending on coverage needs, all libraries can be pooled for one run.The amplicons from each reaction mixture were

pooled in equimolar ratios based on their concentration. Sequencing was performed using the Illumina MiSeq system (Illumina MiSeq, USA), according to the manufacturer’s instructions.

After sequencing, The two short Illumina readings were assembled by PEAR software(version 0.9.8) according to the overlap and fastq files were processed to generate individual fasta and qual files, which could then be analyzed by standard methods. The effective tags were clustered into operational taxonomic units (OTUs) of ≥97% similarity using Usearch software(version 11.0.667). Chimeric sequences and singleton OTUs (with only one read) were removed, after which the remaining sequences were sorted into each sample based on the OTUs.The tag sequence with the highest abundance was selected as a representative sequence within each cluster. Bacterial and fungal OTU representative sequences were classified taxonomically by blasting against the RDP Database and UNITE fungal ITS Database, respectively.
